# Designing and Evaluating IT Applications for Informal Caregivers: Scoping Review

**DOI:** 10.2196/57393

**Published:** 2024-10-23

**Authors:** Shweta Premanandan, Awais Ahmad, Åsa Cajander, Pär Ågerfalk, Lisette van Gemert-Pijnen

**Affiliations:** 1 Department of Informatics and Media Uppsala University Uppsala Sweden; 2 Division of Visual Information and Interaction Department of Information Technology Uppsala University Uppsala Sweden; 3 Department of Psychology, Health, and Technology Faculty of Behavioral, Management and Social Sciences University of Twente Enschede Netherlands

**Keywords:** burnout, caregiver, design guidelines, design recommendations, evaluation, health care services, implementation, informal caregiver, long-term care, mobile app, facilitators, barriers, usability, work-life balance

## Abstract

**Background:**

Informal caregivers, often family members or friends, play a crucial role in supporting individuals with chronic illnesses, disabilities, or age-related needs. However, the demands of caregiving can be overwhelming, leading to stress, burnout, and negative impacts on caregivers’ well-being. IT applications have emerged as potential solutions to support informal caregivers, but their design and evaluation often lack a comprehensive understanding of caregivers’ needs and preferences. By understanding caregivers’ perspectives on these issues, this review aimed to inform the development of more effective and user-centered IT solutions that truly support caregivers’ needs.

**Objective:**

The purpose of this study was to conduct a scoping review to outline design recommendations for IT applications gathered from informal caregivers. In addition, this study presents evaluations of the use of IT applications by informal caregivers.

**Methods:**

A five-step scoping review methodology was used to map relevant literature in the following manner: (1) research question identification, (2) relevant study identification, (3) selection of pertinent studies for review, (4) data charting from the selected literature, and (5) summarization and reporting of results. A structured search was conducted across the PubMed, Scopus, IEEE Xplore Digital Library, Web of Science, and ACM Digital Library databases. In addition, reference list hand searches and keyword searches in Google Scholar were undertaken. The inclusion criteria comprised research articles (journal and conference) focusing on IT applications tailored for informal caregivers, primarily qualitative studies. Two reviewers independently identified articles for review and extracted the data. Conflicts were resolved through discussion, with a third reviewer consulted if consensus could not be attained. Thematic analysis was used to analyze the data.

**Results:**

A total of 82 articles were selected for the scoping review—44 (54%) related to design and 39 (48%) related to evaluation of IT applications. Six overarching themes emerged related to designing IT applications: information access, support systems, streamlining care-related tasks, fostering informal caregivers’ well-being, communication with formal health care professionals, and task support. Furthermore, 3 broad themes emerged related to the evaluation of IT applications for informal caregivers: facilitators of using IT applications, barriers to using IT applications, and suggestions for improving IT applications for informal caregivers.

**Conclusions:**

To our knowledge, this is the first study to map the literature on the design and evaluation of IT applications for informal caregivers. This scoping review outlines current practices and recommendations for designing and evaluating the use of IT applications for informal caregivers. It identifies 6 key design themes and 3 evaluation themes, offering valuable insights for future development in this field. These findings provide a road map for enhancing user-centric IT solutions in informal caregiver support technologies.

**International Registered Report Identifier (IRRID):**

RR2-10.2196/47650

## Introduction

### Background

The demand for informal caregiving is increasing due to reduced hospital and nursing home resources, an aging population, and rising disability rates [1]. Informal caregivers, hereafter referred to as caregivers, are friends or family members who offer care to individuals dealing with illness or disability [1,2]. Informal caregivers provide their relatives with essential care, including practical support and nursing care, saving society considerable expenses [3,4]. The motivations for providing informal care are often rooted in personal and relational factors, such as reciprocity, affection, and family values [5]. However, this role can significantly impact their employment, health, and family life [6]. Caregivers often lack information and support and have unmet needs for respite, financial assistance, and recognition [7,8]. Despite these challenges, informal caregivers are often the only option for care when formal services are not available [9]. However, many informal caregivers do not receive adequate support or assistance [10].

Some informal caregivers report positive experiences from their caregiving journey, including personal accomplishment, strengthened relationships, and personal growth [11,12]. These positive aspects are influenced by factors such as good medical counseling, family and friends’ support, and effective cognitive-emotional regulation [11,12]. However, they also face challenges, such as emotional and social aspects, loneliness, and caregiving burdens [11,13,14]. The intensity of caregiving and the presence of other responsibilities can impact their happiness [15]. Moreover, female caregivers in particular often provide more intensive and complex care, leading to poor emotional health [16]. Despite these challenges, informal caregivers can experience personal growth and resilience, which may help them sustain their support over time [14]. However, there is a care gap that means that the need for informal caregivers is rapidly increasing but the pool of potential informal caregivers is shrinking [17]. The existence of this care gap highlights the need to explore IT applications to support caregivers.

### IT Applications for Informal Caregivers

IT applications for informal caregivers are designed to provide support, resources, and assistance to individuals caring for family members or friends with chronic illnesses, disabilities, or aging-related issues. These applications can range from mobile apps to web-based platforms and aim to streamline the process of building an informal care team, provide educational information, help remember physicians’ appointments, and coordinate care among all involved parties [18]. IT applications are beneficial for informal caregivers in various ways. They can provide access to information and support, reduce social isolation, and improve caregivers’ and care recipients’ quality of life [18-20]. Various IT applications have been developed to support informal caregivers’ caregiving activities. These applications include mobile apps that provide information, resources, and solutions to common problems [19] and web-based platforms that offer knowledge about caring and facilitate the formation of support networks [21]. Smartphone apps have also been designed to deliver personalized health information to caregivers of patients with chronic illnesses [22]. However, deploying these solutions is not without challenges, including technology-related, organizational, socioeconomic, and ethical issues [23]. Information, comprehension, motivation, time available, perceived burden, and perceived informal caregiving competency have been identified as factors influencing technology adoption and use by informal caregivers [24]. Hence, it is recommended that designers involve informal caregivers in the design process, build their digital skills, and raise awareness of the potential benefits of IT solutions [23]. Despite these challenges, the potential of IT applications to support informal caregivers is notable, and further research is needed to explore their design and use on a broader scale [22,25]. There is a need to consolidate existing knowledge on design recommendations for IT applications for caregivers. This would offer designers a valuable starting point when developing new solutions, serving as a comprehensive resource. In addition, it would provide an overview of current evaluations of IT applications for informal caregivers, helping designers understand which features are valued and which are less effective from the caregivers’ perspective.

There are some reviews in the field of IT applications for informal caregivers in the recent literature. Hassan [23] identified challenges in deploying information and communications technology solutions, including technological, organizational, socioeconomic, and ethical issues. Sala-González et al [19] found that mobile apps for caregivers often address their needs but the evidence of their effectiveness is limited. Furthermore, Romero-Mas et al [26] highlighted the potential of virtual communities of practice in supporting informal caregivers. Krick et al [27] emphasized the need for high-quality evaluations of digital technologies for caregivers. Hence, a review of current practices in designing and evaluating the use of IT applications for informal caregivers is crucial due to the increasing role of informal caregivers in health care and the potential of IT applications to support them [18]. Table 1 provides an overview of recent review studies in this area. Through our scoping review, we contribute to the existing literature on IT applications for informal caregivers by identifying and summarizing knowledge about design recommendations and evaluation of the use of IT applications to support informal caregivers.

**Table 1:** Overview of recent review studies.

Study, year	Review study aims	Articles reviewed, N
Hassan [23], 2020	Identify challenges in deploying ICT^a^ solutions, including technological, organizational, socioeconomic, and ethical issues	31
Sala-González et al [19], 2021	Evaluate current mobile apps for informal caregivers and assess whether they were developed considering the needs of caregiver users	11
Romero-Mas et al [26], 2020	Highlight the potential of virtual communities of practice in supporting informal caregivers	46
Krick et al [27], 2019	Review digital technologies in both informal and formal care settings focusing on their AEE^b^	715
Martínez-Alcalá et al [28], 2016	Identify the opportunities that ICT offers to health services, specifically for patients with dementia and their families	26
Powell et al [29], 2008	Understand the effectiveness of networked ICT interventions in supporting carers of people with dementia	15
Guessi Margarido et al [22], 2022	Investigate the nature and extent of native smartphone apps for informal caregivers of patients with chronic illnesses	36
Meyer et al [30], 2018	Conducted a systematic review of novel technology as a platform for interventions for caregivers and individuals with severe mental illnesses	11

^a^ICT: information and communications technology.

^b^AEE: acceptance, effectiveness, and efficiency.

### Objectives

The purpose of this study was to conduct a scoping review of current practices and recommendations for designing and evaluating the use of IT applications by informal caregivers. Design recommendations for IT applications are best practices and guidelines that designers can use to create user-friendly, efficient, and effective applications. For instance, a design recommendation could involve providing support groups to informal caregivers, ensuring that the application can provide an online forum or space for caregivers to share their experiences and have a sense of community.

We also provide a summary of evaluations of the use of IT applications for informal caregivers. Although many applications for caregivers are designed based on identified needs through interviews and focus groups, the existing literature on this topic has not been compiled comprehensively. The research questions (RQs) that this scoping review addressed were as follows:

1. What are the design recommendations for developing IT applications for informal caregivers? (RQ 1)
2. How is the use of IT applications evaluated by informal caregivers? (RQ 2)

## Methods

### Overview

The methodological framework by Arksey and O’Malley [31] for scoping reviews was followed for this review. The framework outlines the following five stages: (1) identification of the RQ; (2) identification of relevant studies; (3) selection of relevant studies; (4) charting the data from the selected literature; and (5) collating, summarizing, and reporting the results. Reporting follows the guidelines defined by the PRISMA-ScR (Preferred Reporting Items for Systematic Reviews and Meta-Analyses extension for Scoping Reviews; Multimedia Appendix 1) [32] checklist. Full details of the methods can be found in our published protocol [18]. There were minor updates from the protocol. The second author conducted the full-text review for a portion of the study. The published protocol originally reported the number of articles from the initial database searches in 2021. However, a subsequent database review was conducted in 2023 along with manual searches. These updated numbers are now reflected in the flowchart.

### Selection Criteria

The literature search was carried out with the support of health psychology information specialists from the University of Twente, and we also had consultations with university librarians at Uppsala University. The selection criteria were defined based on the population, intervention, comparator, outcome, and study design (PICOS) approach [33].

Informal adult caregivers, typically family members, partners, or friends aged >18 years, provide unpaid care for individuals with long-term illnesses or disabilities. This review included studies focusing on informal caregivers and excluded those focused on care recipients, formal caregivers, or health care professionals. The interventions considered were IT applications aimed at informal caregivers, ranging from mobile apps to web platforms providing assistance, resources, and support. Studies on applications targeting both care recipients and informal caregivers were included if the caregivers’ role was substantial. IT applications designed for care recipients but influenced by informal caregivers’ opinions were excluded.

As this review did not compare studies with other treatments, a comparator was not applicable. The included studies focused on designing or evaluating IT applications for caregivers or both. Design-related papers covered caregivers’ needs and preferences for web-based applications or interventions, whereas evaluation papers addressed usability, evaluation, assessment, feasibility, and user experience. All study designs were eligible except descriptive or quantitative studies on system adoption based on theoretical models. Studies on IT applications targeting patients, even if developed with input from caregivers, patients, and health care professionals, were also excluded.

During the screening, the reviewers used the following inclusion and exclusion criteria. The inclusion criteria were (1) conference articles, journal articles, and early-access articles; (2) studies on usability tests of IT applications or platforms; (3) studies describing user needs or requirements for IT applications for informal caregiving; (4) studies that described the evaluation of IT applications focusing on what works or what is preferred, along with user experience; (5) studies describing the development process of IT applications for informal caregiving; and (6) studies on IT applications to train informal caregivers in their caregiving activities. The exclusion criteria were (1) review articles, editorial and opinion papers, news articles, books and book chapters, theses, and clinical case papers; (2) descriptive or quantitative studies assessing the adoption of a system or criteria for adoption based on a theoretical model; (3) studies focused on care recipients and formal caregivers; and (4) studies on IT applications targeted at patients but developed based on interviews with patients and informal caregivers along with health care professionals.

### Search Strategy

Electronic database searches were conducted in PubMed, the IEEE Xplore and ACM digital libraries, Scopus, and Web of Science on metadata such as titles, abstracts, and keywords.

The literature search strategy was constructed based on terms related to the following PICOS criteria: (1) informal caregivers (eg, *caregiver*, *spouse*, and *partner*), (2) IT applications (eg, *internet*, *app*, or *eHealth*), and (3) design and evaluation (eg, *design*, *evaluation*, *needs*, *usability*, or *experience*). Librarians were consulted when constructing the search string. Textbox 1 provides an overview of the different groups of keywords. There were no date restrictions. Complete search strategy is available in Multimedia Appendix 2.

**Keywords for the search string.**

**Group 1: informal caregivers**

“Home nursing” OR “informal care*” OR “family care*”

**Group 2: IT solutions**

“Mobile application*” OR “ICT solution*” OR “ICT” OR eHealth OR “e-coaching system*” OR “*coaching system*” OR “digital solution*” OR “IT solution*” OR “internet-based interventions*” OR telehealth*

**Group 3: design or evaluation**

Design OR evaluation OR effectiveness OR usability OR requirements OR needs OR perspective OR “user experience*”

The secondary search strategy included conducting forward and backward citation screening for all included studies. In addition, we conducted manual searches in Google Scholar using keywords. The Google Scholar search was conducted using the keyword field in Publish or Perish (version 8.9.4538) [34]. Multiple combinations using keywords from each search group were used for the Google Scholar search on metadata, and 4 combinations yielded the most relevant results. Each of the following search strings was searched separately, and 100 hits were collected from each search: (1) *family carer design OR evaluation “internet intervention,”* (2) *family carer design OR evaluation ICT*, (3) *family carer design OR evaluation “digital intervention,”* and (4) *family carer design OR evaluation application*.

### Study Selection

The initial review process (as seen in the protocol [18]) was carried out in November 2021. Search results were first deduplicated [35] and stored using EndNote X9 (Clarivate Analytics) [36]. A total of 428 deduplicated records were imported into Rayyan (Rayyan Systems Inc) [37], where title and abstract screening was carried out by 1 reviewer (SP). Full-text screening of the initial results was conducted independently by 2 reviewers (SP and AA) using the established criteria. Conflicts during the screening process were resolved through discussion, and a third reviewer (ÅC) was consulted as needed. Records retrieved from an updated search for papers published between October 2021 and November 2023 (537 deduplicated records) were screened by 1 reviewer (SP). Secondary searches were conducted by a librarian. For these records, title and abstract screening and full-text review were conducted by 1 reviewer (SP). Abstracts, editorial and opinion papers, news articles, books and book chapters, theses, and clinical case papers were excluded as these sources lacked rigor. Reviews and protocols were not included; however, published results of relevant protocols were sought. In addition, unpublished results of relevant protocols were sought from the authors if published results were not yet available.

### Data Extraction

Details from the included records, such as authors, publication year, study purpose, study location, study design, and condition cared for, were extracted by the first reviewer (SP) using a Microsoft Excel spreadsheet (version 2021; Microsoft Corp) and confirmed to be accurate and complete by the second reviewer (AA). Each record was classified as either a design or evaluation study. The articles were imported into the NVivo software (version 12; Lumivero). A table with a summary of the included articles can be found in Multimedia Appendix 3 [8,38-118].

### Data Analysis

This study used the NVivo software (version 12) to gather and systematically structure study data. Full-text articles were imported as PDF files into NVivo for data extraction, analysis, and coding. Analysis followed an iterative method involving combining, categorizing, summarizing, and comparing information across studies. Relevant data were identified and categorized under broader emergent themes related to each RQ. As Braun and Clarke [119] outlined, inductive thematic analysis was applied to analyze texts from the included studies. Identifiable design recommendations and perceptions of evaluations of the use of IT applications by informal caregivers were extracted from analyzing the included articles, forming overarching themes. Any design recommendations and evaluations of use not fitting into established themes were iteratively added as new themes [120,121]. Two reviewers (SP and AA) conducted the formal thematic analysis of the included articles involving coding and identifying themes. Afterward, they met to discuss their findings, and through this discussion, finalized the themes. As their initial themes were largely aligned, the discussion helped solidify the results, ensuring the trustworthiness and validity of the findings. An expert reviewer (ÅC) with extensive experience in this area performed a final review of the codes and themes. The expert also reviewed the list of included articles to ensure that no significant studies were overlooked.

## Results

### Overview

**Figure 1:**
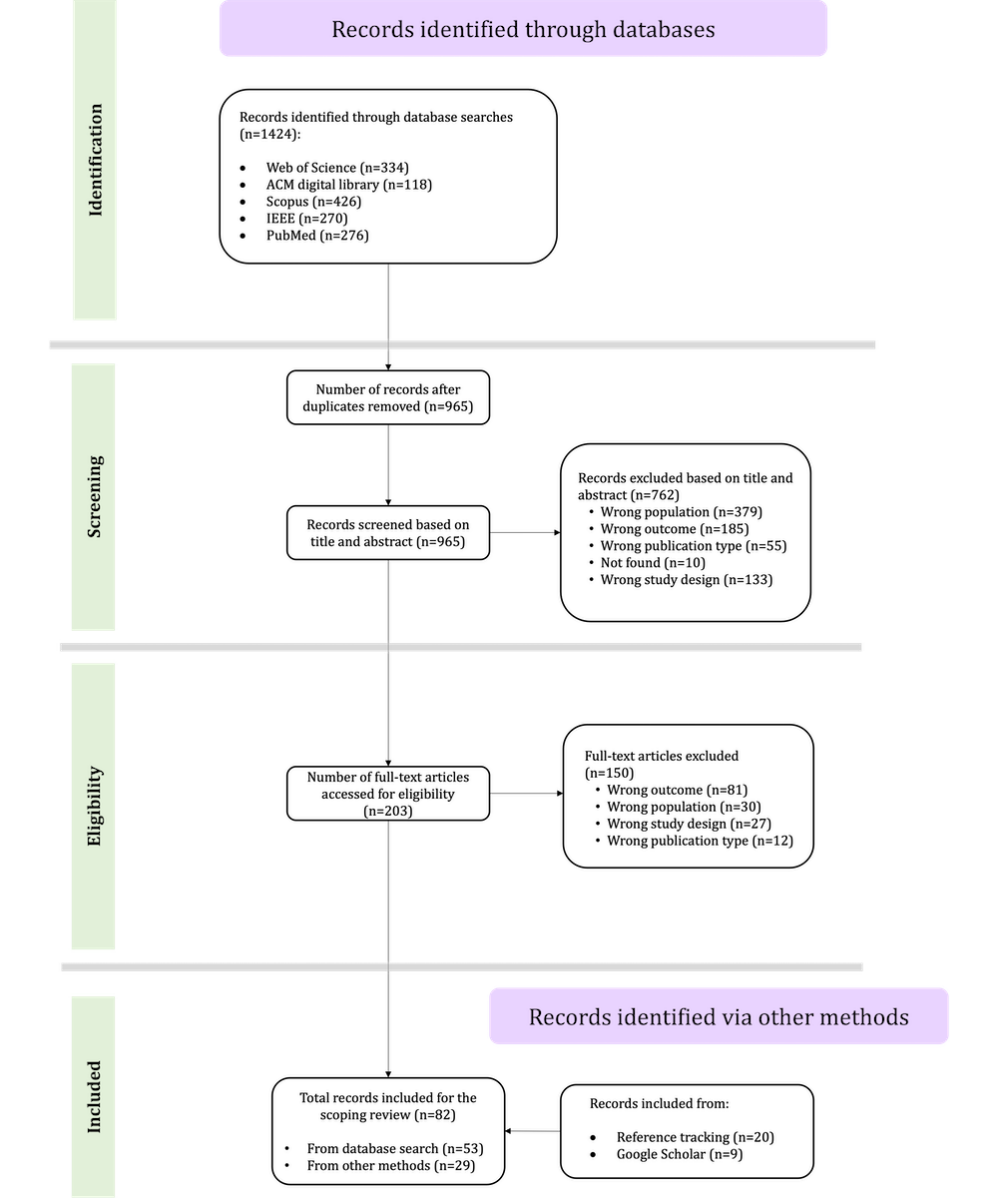
Study selection flow diagram.

The database searches yielded 1424 records (Figure 1). After duplicate records were removed (459/1424, 32.23%), titles and abstracts (965/1424, 67.77%) were screened before full texts (203/965, 21%) were retrieved for eligibility screening. A total of 29 of the included reports were identified using secondary search strategies, namely, reference tracking (n=20, 69%) and manual keyword searches in Google Scholar (n=9, 31%). In total, 82 reports were included in the study.

First, results for designing IT applications, which addresses the first RQ, are presented. Of the 82 included articles, this study reviewed 44 (54%) with design recommendations for IT applications supporting informal caregivers. A total of 6 main themes emerged: optimizing information access, support systems for informal caregivers, streamlining care-related tasks for informal caregivers, fostering informal caregivers’ well-being, communication with formal health care professionals, and task support. Most of the included studies on design used qualitative methods, including interviews (20/44, 45%) and focus groups (10/44, 23%) with caregivers, whereas some also used mixed methods (5/44, 11%). Some of the included design studies (14/44, 32%) were focused on dementia caregivers, but we also found a mix of informal caregivers for cancer (5/44, 11%), older adults (8/44, 18%), and children with medical complexities (4/44, 9%).

Thereafter, the findings of evaluations of use of IT applications, which addresses the second RQ, are presented. The analysis of 48% (39/82) of the studies revealed 3 main themes: facilitators of using IT applications, barriers to using IT applications, and suggestions to improve IT applications. All studies indicated that IT applications had a positive reception among informal caregivers. Most evaluations (28/39, 72%) were conducted through qualitative interviews, with some using mixed methods (11/39, 28%). Qualitative approaches provided detailed insights into caregivers’ needs, whereas some medical studies (4/39, 10%) used randomized controlled trials to assess the impact of IT applications on caregivers and patients. Although many studies (18/39, 46%) focused on mental impairments, there was a research gap concerning caregivers managing physical diseases, highlighting an area for future study.

### Design Recommendations for IT Applications

#### Overview

**Figure 2:**
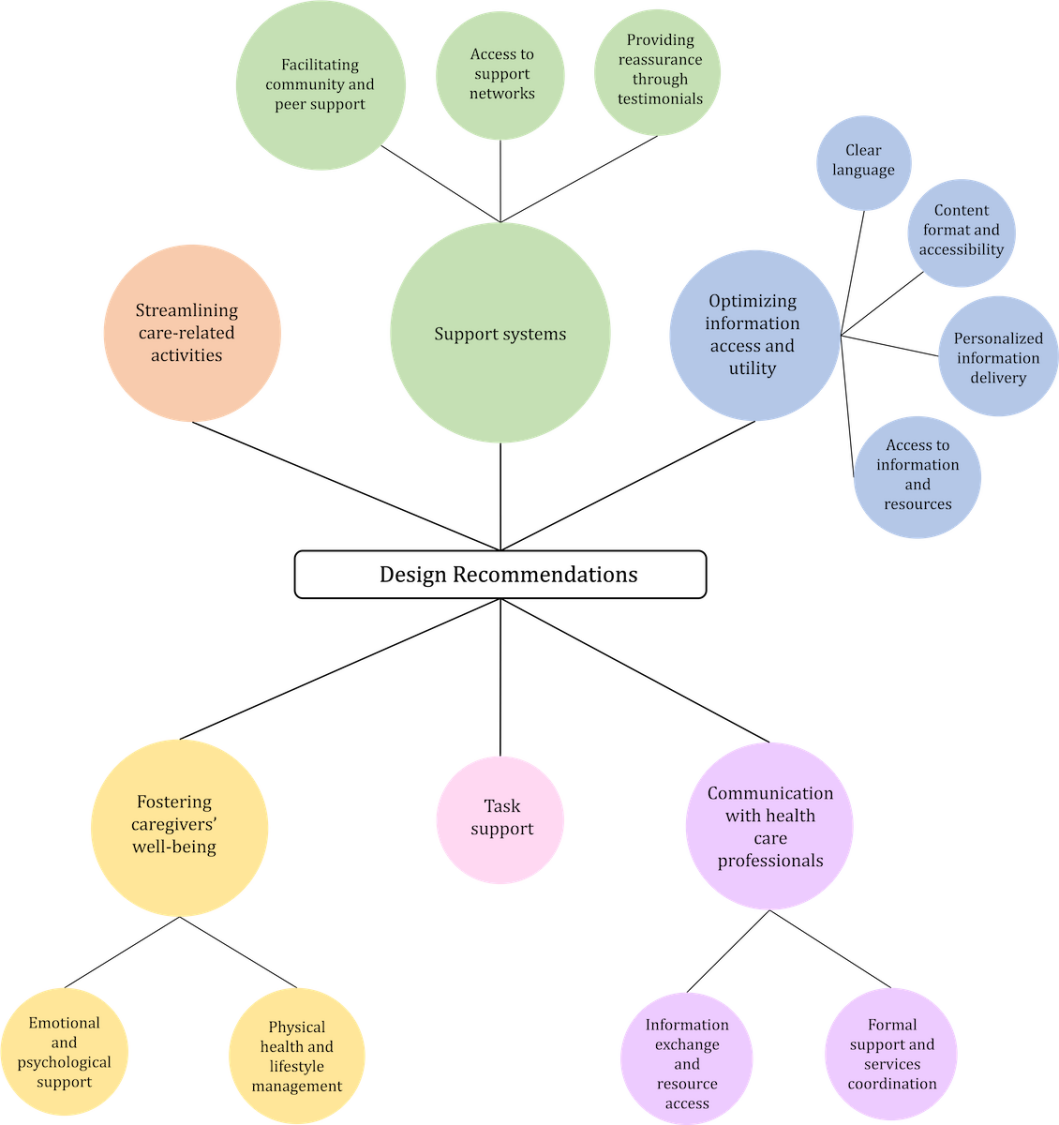
Bubble chart illustrating the number of articles in each theme and subtheme for the design recommendations for IT applications.

In this section, we present 6 themes and several subthemes of design recommendations for IT applications for informal caregivers. A summary of all the themes and their corresponding subthemes is presented in Table 2. Figure 2 illustrates the number of articles in each theme and subtheme in the form of a bubble chart.

**Table 2:** Summary of themes and subthemes for the design recommendations for IT applications with references.

Theme and subtheme			References
**Optimizing information access and utility**			
	Access to information and resources	Premanandan et al [8], Rathnayake et al [38], Dickman Portz et al [42], Heynsbergh et al [45], Tixier and Lewkowicz [49], McHugh et al [53], Ahmad et al [57], Premanandan et al [58], Ciuffreda et al [60], Haji Mukhti et al [62], Fan et al [63], Egan et al [64], Köhle et al [68], Masterson-Algar et al [70], Moreno-Cámara et al [71], Sepehri et al [72], Siddiqui et al [74], Williamson et al [76], and Liverpool and Edbrooke-Childs [77]	
	Personalized information delivery	Nurgalieva et al [43], Hwang et al [54], Moberg et al [56], Premanandan et al [58], Egan et al [64], and Ducharme et al [79]	
	Content format and accessibility	Ahmad et al [57], Molinari-Ulate et al [59], Masterson-Algar et al [70], and Shreve et al [73]	
	Clear language	Molinari-Ulate et al [59], Masterson-Algar et al [70], and Xu et al [78]	
**Support systems**			
	Facilitating community and peer support	Rathnayake et al [38], Lobão et al [40], Leslie et al [41], Dickman Portz et al [42], Heynsbergh et al [45], Allemann et al [47], Tixier and Lewkowicz [49], Bosch and Kanis [51], Meiland et al [52], McHugh et al [53], Renyi et al [55], Ahmad et al [57], Premanandan et al [58], Molinari-Ulate et al [59], Fan et al [63], Hashemi et al [67], Köhle et al [68], Moreno-Cámara et al [71], Shreve et al [73], Siddiqui et al [74], Vaughan et al [75], and Xu et al [78]	
	Access to support networks	Nurgalieva et al [43], Heynsbergh et al [45], McHugh et al [53], Renyi et al [55], and Egan et al [64]	
	Providing reassurance through testimonials	Premanandan et al [8], McHugh et al [53], Ahmad et al [57], Premanandan et al [58], and Giroux et al [65]	
**Streamlining care-related tasks**			Rathnayake et al [38], Macaden et al [39], Lobão et al [40], Chaar et al [46], Meiland et al [52], Premanandan et al [58], Ciuffreda et al [60], Gris et al [61], Haji Mukhti et al [62], Egan et al [64], Gutierrez and Ochoa [66], Sepehri et al [72], Williamson et al [76], Liverpool and Edbrooke-Childs [77], and Xu et al [78]
**Fostering caregivers’ well-being**			
	Emotional and psychological support	Lederman et al [44], Heynsbergh et al [45], Chaar et al [46], Bosch and Kanis [51], Ahmad et al [57], Fan et al [63], Hashemi et al [67], Moreno-Cámara et al [71], Sepehri et al [72], Shreve et al [73], Xu et al [78], and Ducharme et al [79]	
	Physical health and lifestyle management	Rathnayake et al [38], Lederman et al [44], Heynsbergh et al [45], Chaar et al [46], McNaney et al [48], Fan et al [63], Hashemi et al [67], Moreno-Cámara et al [71], Sepehri et al [72], Shreve et al [73], Xu et al [78], and Ducharme et al [79]	
**Communication with formal health care professionals**			
	Information exchange and resource access	Heynsbergh et al [45], Allemann et al [47], Schorch et al [50], Premanandan et al [58], Molinari-Ulate et al [59], Fan et al [63], and Giroux et al [65]	
	Formal support and service coordination	Premanandan et al [58], Moreno-Cámara et al [71], Shreve et al [73], and Ducharme et al [79]	
**Task support**			Premanandan et al [8], Meiland et al [52], Moberg et al [56], Molinari, Ulate et al [59], Fan et al [63], Egan et al [64], Gutierrez and Ochoa [66], and Masterson-Algar et al [70]

#### Theme 1: Optimizing Information Access

##### Overview

This theme includes design recommendations with regard to providing information access to informal caregivers to help them in their caregiving role. A total of 59% (26/44) of the articles reviewed were classified under this theme, and most (19/44, 43%) referred to information access for informal caregivers. This includes education and training opportunities tailored to informal caregivers’ needs, facilitating access to diverse resources and services, delivering personalized information, ensuring enhanced content formats and accessibility, and emphasizing clear language and clarity in communication.

##### Access to Information and Resources

In the literature reviewed, IT applications provided access to training opportunities to assist informal caregivers in developing their skills for effective caregiving [38,71]. Tailored resources, such as educational materials, classes, and programs were integrated to offer practical support and guidance [63,64]. Some applications also included provisions to facilitate advanced care planning as a crucial element in caregivers’ routines [42]. In addition, these applications facilitated access to peer-reviewed research, recognizing the importance of evidence-based insights and strategies for caregivers [63,64]. IT applications for informal caregivers had quick access to relevant links and references [45,53,62,68,70,72,74]. They facilitated the establishment of a common knowledge base among caregivers, fostering a shared understanding and effective care strategies [49,60]. In addition, these applications offered guidance on accessing formal services such as professional caregivers or housekeeping [63], acknowledging the importance of aiding informal caregivers in navigating available resources. Some IT applications also reported providing access to medical health records [76], which is important for informal caregivers in delivering comprehensive care. A few of these applications also had tailored web-based resources [53,62,72,77] specifically aimed at informal caregivers. These web-based resources may include materials such as information articles, guides, tutorials, or forums that provide information, tips, and assistance relevant to the needs and challenges faced by informal caregivers.

##### Personalized Information Delivery

IT applications also prioritized delivering tailored information specific to caregivers’ unique situations [43,64]. Customizing information for caregivers consistently assisted them in their daily caregiving responsibilities [54]. In addition, these applications featured relevant personalized information addressing caregivers’ individual needs and challenges [43,56,64,79]. This tailored approach regarding IT applications emphasizes that caregivers prefer personalized over generic information. For instance, caregivers can benefit from personalized information based on the condition that they provide care for, their relationship with the relative, the stage of caregiving, location, and more. Furthermore, the emphasis on personalized information within these applications is indicative of the diverse and ever-changing nature of caregiving responsibilities. This is because the type of information and support may change based on the progression of the condition for which care is provided.

##### Content Format and Accessibility

Various articles reviewed (4/44, 9%) discussed IT applications that emphasized delivering content to informal caregivers in varied formats, including video, audio, images, and snippets [59]. In addition, content in short text format was particularly valuable for informal caregivers as they preferred short and easily understandable information [70]. Moreover, the studies stressed the importance of illustrations and appropriate visuals [70] in aiding comprehension and engagement among informal caregivers. The dynamic information needs of informal caregivers require diverse content formats to accommodate their evolving requirements, ensuring accessibility and relevance to their caregiving tasks [73].

##### Clear Language

Numerous studies (3/44, 7%) stressed the importance of using good, easy, and appropriate language in content targeted at informal caregivers [59,70]. Simplicity and clarity are key, along with the need to avoid jargon that might hinder effective comprehension of information by caregivers [59,70]. Furthermore, the studies also indicated that a few applications focused on debunking illness myths [78], which is important for shaping caregivers’ understanding. In addition, caregivers actively sought knowledge and a comprehensive history of the illnesses they provide care for [78].

#### Theme 2: Support Systems for Informal Caregivers

This theme presents recommendations that pertain to providing support structures for informal caregivers in the form of online forums, peer support, and social support. It also includes the provision of testimonials.

##### Facilitating Community and Peer Support

The functionalities required by caregivers from IT applications to facilitate community and peer support involved a diverse set of tools aimed at fostering connections and shared experiences among informal caregivers. In the 59% (26/44) of reviewed articles that were classified under this theme, these functionalities prioritized creating a sense of community and shared experiences [57,58], allowing caregivers to relate to and connect with others in similar situations. Peer support is crucial, enabling caregivers to gain insights and emotional reinforcement from their peers [41,42,53,58].

Moreover, the granularity of the information exchanged should also be considered, allowing caregivers to exchange comprehensive experiences and connect with peers [49]. Enabling the sharing of experiences among caregivers becomes vital for building social ties [38,40,47,55,68,74] and solidarity within the caregiving network [51-53]. Feedback from peer groups becomes an invaluable resource, providing diverse perspectives and guidance to caregivers facing similar challenges [59,63,67,71,73,75,78].

Engagement in dedicated caregiver groups [75] and the provision of virtual meeting spaces [57,75] were important functionalities, offering platforms for interactions and support irrespective of geographical limitations. This engagement could be in terms of reading other caregivers’ experiences, asking questions, answering questions, venting their frustrations, or just speaking with other caregivers in similar situations. In addition, facilitating face-to-face group engagements [45] and providing access to online forums [57,58] such as Facebook groups satisfies varying preferences, allowing caregivers to seek and extend support.

##### Access to Support Networks

Improving access to support networks is crucial for the well-being of informal caregivers.

The capability to reach or access a care network is essential as it allows caregivers to connect with relevant support systems [43,53]. Finding peers and gaining access to social contacts of fellow caregivers enable the expansion of one’s support network, fostering connections and shared experiences [45].

The inclusion of a Pinboard feature serves as a valuable resource, allowing caregivers to gather and share pertinent information and resources [55]. Access to social media and messaging platforms becomes crucial, providing channels for communication and information exchange among caregivers [64]. In addition, having access to an interaction book [72] offers a centralized space for storing and sharing essential caregiving-related information.

##### Providing Reassurance Through Testimonials

This recommendation of providing reassurance through testimonials is crucial as it serves as a means to offer confidence and affirmation to caregivers in their caregiving journey. IT applications provide caregivers with reassurance, instilling a sense of trust and confidence [53]. In addition, providing a space for caregivers to incorporate useful testimonials regarding caregiving experiences becomes essential as it can offer insights and real-life experiences to guide fellow caregivers regarding available support services or useful tips [65].

#### Theme 3: Streamlining Care-Related Tasks for Informal Caregivers

This theme emphasizes design recommendations specifically to assist with caregiving tasks. It includes features for monitoring medication, tracking the care recipient’s condition, and streamlining care coordination. A total of 34% (15/44) of the articles reviewed were classified under this theme. In addition, these designs include comprehensive support for daily tasks, offering tailored assistance that caters to the unique needs of caregivers and care recipients.

Some IT applications had a provision for caregivers to record and document caregiver problems [38]. This feature can facilitate effective communication between caregivers and health care professionals, ensuring that the needs and concerns of the caregivers are adequately addressed. Effective illness management is a critical caregiving task, with IT applications offering features that assist caregivers in handling the health needs of their care recipients [38]. This may include tools for tracking symptoms, medication management, and accessing relevant health information. Continuously monitoring and tracking health parameters becomes integral for assessing progress and adjusting interventions as needed, ensuring the best health outcomes for the person under care [39,52,58,60,78]. Many applications also provided support for daily activities and access to services and facilities that can aid caregivers in their responsibilities [38]. This may encompass scheduling assistance, access to community resources, and information about support services available to caregivers and care recipients. Incorporating information and resources related to using care equipment was another useful application feature [38]. This may involve guidance on selecting and using appropriate care equipment and information on where to access such equipment. Some applications also supported caregivers in effectively managing the care of their patients, providing tools for organizing and coordinating care tasks [40]. This may include features for care coordination, communication with health care providers, and managing appointments and treatments. Features related to appointment management, treatment tracking, and medication management were essential in IT applications for informal caregivers [40]. These features can help caregivers stay organized and ensure that care recipients receive medical attention and treatments. Some applications also supported caregivers in learning and adapting to new caregiving routines [46]. This may involve providing educational resources, tips for managing changing care needs, and guidance on adapting to evolving caregiving responsibilities. Incorporating features that provide support in emergencies was important for applications for informal caregivers [52]. This may include emergency contact information, guidance on handling emergencies, and access to emergency resources. Many applications also included provisions for managing various care activities, including household tasks, personal hygiene, mobility support, medical care, and care coordination [61]. These features can help caregivers effectively organize and manage their caregiving responsibilities. Some caregivers were involved in physiotherapy activities for themselves and their care recipients, and hence, those applications included resources and guidance on technical skills related to physiotherapy [62]. This may include instructional materials, exercise demonstrations, and information on physiotherapy techniques. Incorporating evidence-based activity plans can be beneficial for caregivers, and the IT applications also provided access to such resources [64]. This may involve evidence-based caregiving strategies, activity recommendations, and guidelines for promoting the well-being of caregivers and care recipients. IT applications also need to include features that allow caregivers to track the evolution of their roles and access contextual information and services accordingly [66]. This may involve documenting changes in caregiving responsibilities, accessing relevant support based on evolving needs, and staying informed about available resources. A caregiving timeline dashboard can be a valuable feature in applications, providing caregivers with a visual representation of caregiving activities and milestones [72,77]. This feature can help caregivers track and manage their caregiving responsibilities effectively. Incorporating features for tracking medications was essential in applications for informal caregivers [76]. This may involve medication schedules, medication administration reminders, and medication use tracking. IT applications should assist caregivers with comprehensive knowledge on medication use and potential side effects [78]. This may include medication information, dosage guidelines, and information on potential adverse reactions. In addition, incorporating resources and information on symptom management was also [78]. This may involve guidance on recognizing and managing symptoms, accessing relevant health care information, and seeking appropriate support.

#### Theme 4: Fostering Informal Caregivers’ Well-Being

##### Overview

This theme presents design recommendations that can assist in fostering informal caregivers’ well-being through IT applications, and 32% (14/44) of the articles reviewed were classified under this theme. There were 2 subthemes, namely, emotional and psychological support and physical health and lifestyle management. This theme presents various recommendations, which include physical, mental, emotional, and social support to ensure that caregivers receive comprehensive assistance and are better equipped to navigate the challenges of caregiving while ensuring their well-being.

##### Emotional and Psychological Support

Emotional and psychological support is essential for caregivers, which includes various well-being features. Focusing on assisting in enhancing informal caregivers’ well-being is crucial, acknowledging their important role [51,57,67]. It is imperative that caregivers feel empowered and have autonomy to enable them to navigate challenges independently and feel effective in their roles [44]. Accessing support opportunities and incorporating mindfulness and counseling was another feature provided in applications to help caregivers manage stress and emotional complexities [45]. The stress of caregiving requires strategies that balance the needs of both the caregiver and the care recipient [46,79]. In addition, some IT applications offered features like positive activity interaction, pleasant activity scheduling, gratitude journaling, and a “positive piggy bank,” all of which can help promote well-being [46]. Encouraging a positive, solution-oriented approach fosters resilience, enabling adaptive coping mechanisms when faced with challenges [51,57]. Using tools such as a life journal becomes beneficial for reflection and self-awareness, aiding in emotional processing and personal growth [72]. Moreover, accessing emotional support through interactive IT applications ensures easy accessibility and engagement, creating a supportive environment [71,73,78]. Encouraging self-care practices acknowledges the importance of caregivers prioritizing their well-being [71]. In addition, integrating mental health support becomes crucial, recognizing and addressing the psychological aspects of caregiving stress [63].

##### Physical Health and Lifestyle Management

Physical health and lifestyle management include various components essential for caregivers’ and care recipients’ well-being. Incorporating entertainment such as music into IT applications becomes a creative way to boost engagement and enjoyment, contributing to caregivers’ physical and emotional wellness [38,48,73]. Sharing medical and well-being data becomes crucial for transparency and collaboration between caregivers and care recipients, reducing misunderstandings and distress while aiding informed decision-making [43]. In addition, focusing on physical well-being through exercise, diet, and nutrition becomes fundamental for promoting healthy lifestyles among caregivers [64,76]. Acknowledging the significance of financial aid as part of health management addresses the broader socioeconomic factors influencing well-being [62,72,79]. Recognizing and appreciating efforts becomes a motivating factor, reinforcing positive behaviors and fostering a supportive environment for caregivers to maintain their well-being [8].

#### Theme 5: Communication With Formal Health Care Professionals

##### Overview

This theme emphasizes the significance of robust communication channels for informal caregivers with formal health care professionals, beginning with comprehensive information about health care facilities. A total of 23% (10/44) of the articles reviewed were classified under this theme. Equipping caregivers with details about available facilities and services aids informed decision-making regarding patient care. Quick access to relevant contacts and references ensures prompt assistance, whereas encouraging interactions between caregivers and health care professionals facilitates necessary guidance and support.

##### Information Exchange and Resource Access

Offering information about hospitals in IT applications provides caregivers essential knowledge, enabling them to take quick action during emergencies [45]. IT applications can be instrumental in sharing critical hospital information, such as location, phone number, and services offered. Caregivers were also interested in accessing hospital-specific navigation in the applications [45]. Providing quick references and details of health care contact persons in the applications can ensure quick assistance when required [45]. Facilitating communication with health care professionals through the applications establishes a crucial link between caregivers and expert advice, supporting informed decision-making [47]. IT applications can help coordinate caregiving and healthcare-related tasks for care recipients by streamlining caregiving responsibilities and improving the efficiency of care provision [50]. In addition, guidance in accessing formal services through IT applications bridges the gap between caregivers and available support systems, ensuring comprehensive care [58]. Caregivers can also benefit from timely feedback from health care professionals using the applications, enhancing the quality of care by incorporating expert advice and recommendations [59]. Leveraging referrals to specialized resource persons using IT applications assists in expanding the support network, providing access to specialized knowledge and guidance [65]. Transparent communication through the IT applications about the care recipient’s health condition becomes crucial for informed decision-making [63]. In addition, guidance in finding appropriate care facilities and accessing facility information using IT applications can contribute to the overall well-being of care recipients [63].

##### Formal Support and Service Coordination

First, formal support, including caregiver assistance and respite care [71,73], provides essential relief and specialized aid to caregivers, ensuring uninterrupted and high-quality care while allowing caregivers crucial moments to relax and recuperate. In addition, receiving help at the right time holds immense significance, guaranteeing assistance precisely when it is most needed, thereby maximizing the efficiency of support services [79].

#### Theme 6: Task Support

A total of 18% (8/44) of the articles reviewed were classified under this theme. The functionalities that were crucial for caregivers in an IT application catering to their needs encompassed a variety of essential features. First, support for memory using calendar functions and reminders becomes crucial, providing organizational assistance to help caregivers manage their schedules and tasks effectively [52]. Integrating supportive dialogues within the system creates a user-friendly and empathetic environment, offering guidance and encouragement when necessary [56]. In addition, providing support for users unfamiliar with IT applications ensures accessibility for individuals with varying levels of technological proficiency [8]. A multi-platform format enhances accessibility, enabling users to use the application across different devices and platforms [59]. Task-based notifications and reminders serve as prompts for caregivers, aiding in task management and ensuring that important activities are not overlooked [63,64]. Incorporating a search feature facilitates quick information retrieval, enabling users to find specific information efficiently. Furthermore, using persuasive strategies [58] within the application can motivate caregivers to engage more effectively with their caregiving tasks [66]. Integrating read-aloud options contributes to accessibility, allowing users with diverse needs or preferences to access information through auditory means [70].

### Evaluation of Use of IT Applications Among Informal Caregivers

#### Overview

**Figure 3:**
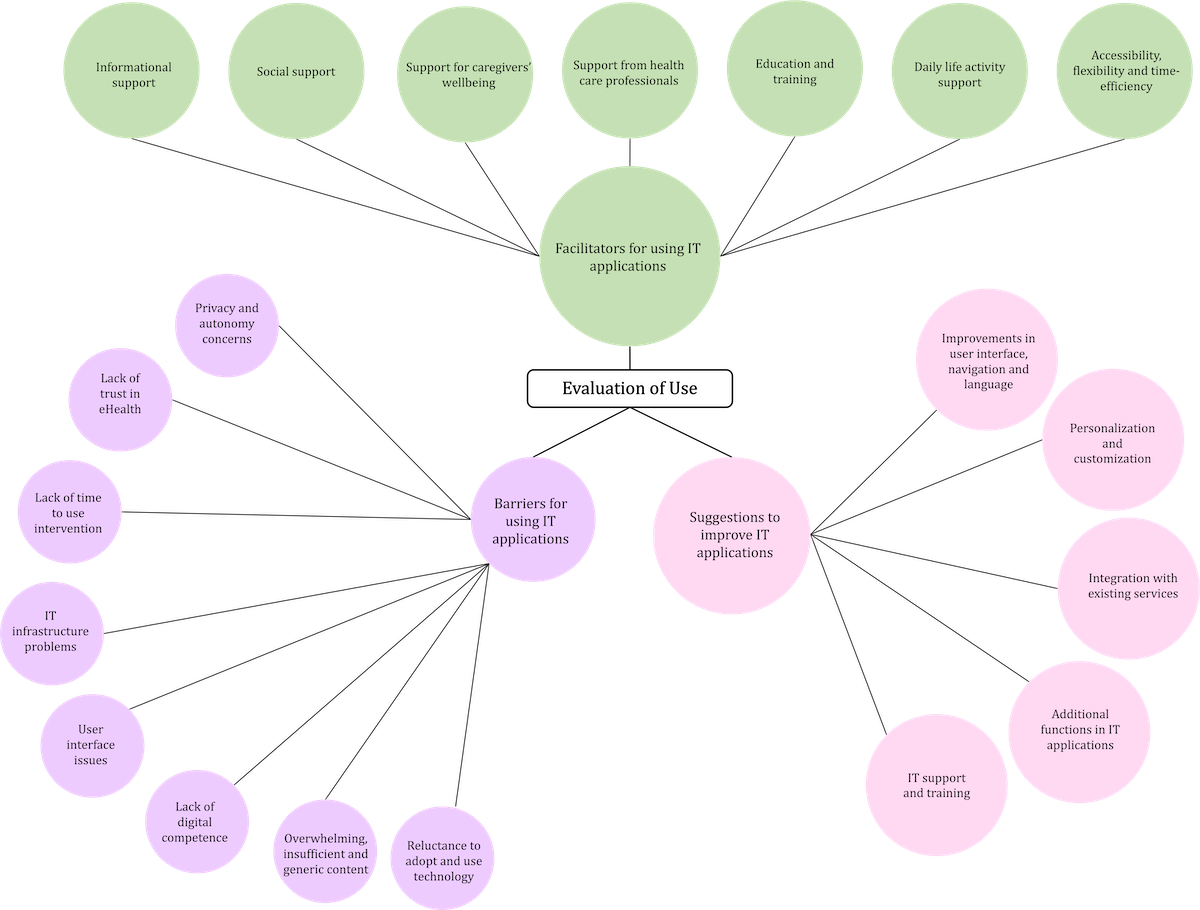
Bubble chart illustrating the number of articles in each theme and subtheme for the evaluation of the use of IT applications.

This section provides an overview of informal caregivers’ perceptions and evaluations of the use of IT applications. The analysis of the selected studies focused on evaluating IT applications designed to support informal caregivers. In total, 3 major themes emerged: facilitators of using IT applications, barriers to using IT applications, and suggestions to improve IT applications. Figure 3 illustrates the number of articles in each theme and subtheme in the form of a bubble chart. A summary of all the themes and their corresponding subthemes is presented in Table 3.

**Table 3:** Summary of themes and subthemes for the evaluation of the use of IT applications with references.

Theme and subtheme		References
**Facilitators of using IT applications**		
	Informational support	Honary et al [117], Rettinger et al [115], Iribarren et al [113], Crotty et al [112], Tibell et al [108], Brown et al [111], Lam et al [105], Husebø [101], Chiu and Eysenbach [98], Andersson et al [93], and Ploeg et al [114]
	Social support	Batchelor et al [116], Ploeg et al [114], Barbabella et al [110], Tibell et al [108], Kwok et al [107], Boutilier et al [106], Hermaszewska and Sin [102], Torp et al [99], Chiu and Eysenbach [98], Blusi et al [96], Lundberg [95], Andersson et al [93], Dam et al [87], Quinn et al [91], Honary et al [117], Blusi et al [97], and Rottenberg and Williams [103]
	Support for caregivers’ well-being	Quinn et al [91], Andersson et al [93], Torp et al [99], Andersson et al [100], Hermaszewska and Sin [102], Boutilier et al [106], Tibell et al [108], Austrom et al [109], Barbabella et al [110], Crotty et al [112], Ploeg et al [114], Batchelor et al [116], and Sin et al [118]
	Support from health care professionals	Lundberg [95], Blusi et al [96], Boutilier et al [106], Austrom et al [109], and Iribarren et al [113]
	Education and training	Wilding et al [88], Gomes et al [90], Brouns et al [92], Kajaks et al [94], Lundberg [95], Chiu and Eysenbach [98], Hermaszewska and Sin [102], Egan et al [104], Austrom et al [109], Brown et al [111], Ploeg et al [114], and Rettinger et al [115]
	Daily-life activity support	Dam et al [87], Chiu and Eysenbach [98], Boutilier et al [106], Tibell et al [108], Brown et al [111], and Ploeg et al [114]
	Accessibility, flexibility, and time efficiency	Lam et al [105], Andersson et al [93], Boutilier et al [106], Austrom et al [109], Brouns et al [92], Blusi et al [96], Kajaks et al [94], and Teles et al [89]
**Barriers to using IT applications**		
	Privacy and autonomy concerns	Dam et al [87], Brouns et al [92], Blusi et al [96], and Honary et al [117]
	Lack of trust in eHealth	Teles et al [89], Barbabella et al [110], and Honary et al [117]
	Lack of time to use the intervention	Brown et al [111] and Ploeg et al [114]
	IT infrastructure problems	Xiao et al [83], Wilding et al [88], Brouns et al [92], Husebø [101], and Lam et al [105]
	User interface issues	Costa Stutzel et al [82], Thompson et al [85], Wilding et al [88], Gomes et al [90], and Lam et al [105]
	Lack of digital competence	Andersson et al [93] and Husebø [101]
	Overwhelming, insufficient, and generic content	Blusi et al [96], Brown et al [111], and Crotty et al [112]
	Reluctance to adopt and use technology	Wilding et al [88], Lam et al [105], and Tibell et al [108]
**Suggestions to improve IT applications**		
	Improvements in user interface, navigation, and language	Renati et al [84], Teles et al [89], Gomes et al [90], Andersson et al [93], Husebø [101], Egan et al [104], Boutilier et al [106], Kwok et al [107], Tibell et al [108], Crotty et al [112], Ploeg et al [114], Honary et al [117], and Sin et al [118]
	Personalization and customization	Cheng et al [81], Renati et al [84], Teles et al [89], Andersson et al [93], Egan et al [104], Boutilier et al [106], Crotty et al [112], Ploeg et al [114], and Honary et al [117]
	Integration with existing services	Brown et al [111] and Batchelor et al [116]
	Additional functions in IT applications	Gomes et al [90], Blusi et al [96], Lam et al [105], Boutilier et al [106], Kwok et al [107], Austrom et al [109], and Ploeg et al [114]
	IT support and training	Wan et al [80], Xiao et al [83], Blusi et al [96], Husebø [101], Barbabella et al [110], Brown et al [111], and Sin et al [118]

#### Theme 1: Facilitators of Using IT Applications

##### Overview

Facilitators of using IT applications emerged as one of the major themes. In this section, we present the facilitators of using IT applications by informal caregivers. A total of 95% (37/39) of the articles reviewed were classified under this theme.

##### Informational Support

Several studies (11/39, 28%) highlighted the usefulness of the information provided by IT applications in assisting caregivers in patient care [93,98,101,105,108,111-115,117]. The studies showed that the information was delivered through various formats, including videos, PDFs, and web pages, covering topics such as patient care, external resources, and the health care system. Caregivers emphasized the value of information on exercise, diet, and medication management, such as handling elevated blood sugar with an endocrinologist’s help [113]. Dementia caregivers appreciated receiving timely, customized information, which helped them understand and cope with illness-related changes and plan for future treatments [114]. The information modules provided quick, accessible advice when needed [115]. Technology-savvy caregivers expressed the need for increased web-based access to accommodate their schedules [113]. Caregivers also valued information on supportive resources, including local support groups and national charities [117]. These resources offered crucial details on medication management and legal rights. Navigating complex medical insurance was a significant challenge, requiring additional support [113]. Another study highlighted the importance of resource links, such as power of attorney information, to help caregivers manage new responsibilities such as financial and health care decisions [114].

##### Social Support

In several studies (17/39, 43%), caregivers consistently highlighted the usefulness and significance of the social support received through various forms in different IT applications [87,91,93,95-99,102,103,106-108,110,114,116,117]. Caregivers appreciated web-based platforms’ interactive services, which connected them with others facing similar challenges [110]. They recommended improving the web-based community-building process to enhance interaction and create a more engaging social environment. Social interaction with those facing similar caregiving challenges was valued as it allowed them to share experiences and find mutual support. Many caregivers found comfort in personal conversations even when problems could not be solved, knowing that they were not alone [117]. While caregivers liked the discussion forums, they suggested promoting popular posts and adding open-ended boards and features such as small chat rooms or focused caregiver cohorts [107].

##### Support for Caregivers’ Well-Being

Caregivers acknowledged the importance of support to improve their well-being, recognizing that the stress associated with caregiving often led to their compromised health and quality of life [91,93,99,100,102,106,108-110,112,114,116,118]. Caregivers acknowledged that the stress of caregiving contributed to physical and psychological issues, affecting their ability to provide care [112]. IT applications encouraged self-reflection, exploration of feelings, care strategies, and goal setting [106,110]. Several studies (6/39, 15%) highlighted that caregivers viewed IT applications as beneficial for psychological well-being, promoting empowerment, competence, and validation [91,93,99,100,114,116]. Using IT applications may also reinforce a sense of competence among working caregivers, helping them meet caregiving demands and see the positive aspects of their situation [93].

##### Support From Health Care Professionals

The possibility of communicating and receiving support from health care providers was highly valued in IT applications [95,96,106,109,113]. The studies indicated that caregivers needed IT applications to include professional support from physicians and nurses along with easy web-based appointment scheduling for tailored health information [110]. They often requested improved communication with health care providers to seek reassurance on patient care decisions [113]. Some IT applications offered videoconferencing with other caregivers and health care professionals, which was highly appreciated [95,109]. Caregivers had positive views on group and video conferencing for support and communication, with studies noting improvements in anxiety, depression, and physical health as a result of these interactions with health care professionals [109].

##### Education and Training

In several IT applications, caregivers found the education and training provided to take care of patients highly useful and helpful for their caregiving responsibilities [88,90,92,94,95,98,102,104,109,111,114,115]. Education and training were provided to caregivers in various forms across different studies. An interesting example involved creating different activities to engage both caregivers and care recipients [115]. Caregivers felt motivated to try out new activities in the applications and adapt them to the personal preferences and possibilities of the relatives they cared for. Participants engaged in activities such as playing ball, watching the news together, decorating the house for Easter, wrapping wool, or looking at photo albums. In addition, most participants expressed satisfaction with the way in which the activities were displayed and described [115].

##### Daily-Life Activity Support

Caregivers participating in various studies found valuable information and functions that aided them in performing daily-life activities [87,98,106,108,111,114]. For instance, a daily care activity checklist was perceived as useful [106]. One study highlighted the effectiveness of using a timeline to organize caregiving activities, with caregivers appreciating a dedicated care book that provided contact details and practical care insights [87]. In addition, information on coping strategies for patients with dementia was valuable for caregivers in managing daily activities and fostering better interactions [98].

##### Accessibility, Flexibility, and Time Efficiency

Caregivers found IT applications to be useful as they offered easily accessible, cost-effective, flexible, and time-efficient solutions. These applications gave caregivers quick access to useful features, ensured smooth functionality, and helped save time during their interactions with other caregivers or healthcare professionals [89,92-94,96,105]. Caregivers found that these IT applications were easy to use and flexible and aligned well with their daily-life activities [105]. One major benefit of IT applications was their accessibility at all times, allowing users to access them when needed and at their convenience in their busy schedules [92]. Caregivers were thankful for customer support as it significantly improved their ability to access and use the IT applications with greater ease and efficiency [106,109].

#### Theme 2: Barriers to Using IT Applications

##### Overview

The theme of barriers to using IT applications explores the challenges faced by informal caregivers in using IT applications for their caregiving tasks. These barriers were privacy and autonomy concerns; lack of trust in IT applications; lack of time to use eHealth interventions; IT infrastructure problems; user interface issues; lack of digital competence; overwhelming, insufficient, and generic content; and reluctance to adopt and use technology. Understanding these barriers is crucial for enabling informal caregivers to effectively leverage IT applications to support their caregiving responsibilities and enhance the quality of care provided to their loved ones. A total of 49% (19/39) of the articles reviewed were classified under this theme.

##### Privacy and Autonomy Concerns

Various studies (4/39, 10%) highlighted caregivers’ concerns about privacy and autonomy [87,92,96,117]. Caregivers expressed apprehension regarding the security of their information on web-based platforms and stressed the importance of increasing their privacy levels [87,117]. Patients viewed therapists’ access to their data not as a breach of privacy but as a motivating factor. However, health care professionals identified internet connections lacking privacy assurances as a barrier to using a web-based platform, emphasizing the necessity for secure data transport [92].

##### Lack of Trust in IT Applications

Several studies (3/39, 8%) highlighted that the lack of trust in the credibility and accuracy of the information provided through IT applications negatively impacted caregivers’ perceptions of their use [89,110,117]. There was extensive discussion about the crucial need for trust in information sources, emphasizing their reliability and legitimacy [89,117]. It is not just the provision of health-related information that matters—establishing trust and credibility is also crucial [117]. Another study identified factors such as a clear layout, interactive features, owner authority, ease of use, and quality content influencing trust in health programs. Caregivers specifically highlighted the importance of layout, owner authority, and content as elements promoting trust [89].

##### Lack of Time to Use the Intervention

Caregivers expressed a strong interest in using the system yet faced difficulties in finding the time to engage with it, attributed to the demanding nature of caregiving and additional responsibilities [111,114]. The challenge is particularly pronounced for overwhelmed caregivers, especially those caring for patients undergoing surgeries or coping with serious conditions, who find it daunting to enter information into the system [111].

##### IT Infrastructure Problems

The challenges in IT infrastructure, such as issues with internet accessibility and hardware, along with technical issues, were a factor hindering the use of IT applications [83,88,92,101,105]. One study highlighted the challenges posed by the lack of consistent internet access, impeding access to health information and engagement in video calls. In addition, hardware issues, particularly those associated with monitoring blood pressure at home using remote devices, were identified as obstacles to effective health monitoring [105]. The absence of reimbursement for IT applications and related infrastructure also posed a barrier for caregivers who could not afford them. Health care professionals cited the lack of reimbursement as a significant obstacle to program implementation due to the associated costs [92].

##### User Interface Issues

Several studies (5/39, 13%) highlighted the problems with user interfaces that affected caregivers’ ease of using the system [82,85,88,90,105]. Caregivers faced several challenges in comprehending the options within the interface, including difficulties understanding specific features such as accessing and using the physical activity function, navigating the notification menu flow, handling alarms, and managing message notifications [82]. They emphasized the need for the interface to provide clear instructions on accessing and using certain functions, underscoring the importance of clarity in using the interface effectively [82,105].

##### Lack of Digital Competence

Many studies (5/39, 13%) emphasized that a lack of digital competence posed significant obstacles for users. These challenges included difficulties in using and navigating digital tools and technologies, struggles in accessing and using web-based information, limitations in effective communication through digital channels, and potential risks related to digital security [83,88,93,101,105]. Especially among older adults, challenges in using IT applications were observed, primarily attributed to insufficient digital competence [93].

##### Overwhelming, Insufficient, and Generic Content

The information provided in the IT applications was overwhelming, too generic, or irrelevant for caregivers, which was perceived as a significant barrier to using IT applications [96,111,112]. Caregivers noted that, especially at the time of diagnosis, they were overloaded with information yet paradoxically felt a need for a personalized search for information [112]. They acknowledged the positive impact of the support received but also underscored additional unmet support needs. Specifically, caregivers expressed receiving support not aligned with their specific requirements, encountering limitations in the availability of support, and feeling that the support provided was structured to meet the provider’s needs rather than addressing the caregivers’ unique needs [96].

##### Reluctance to Adopt and Use Technology

Reluctance to adopt and use technology was evident in studies in which caregivers expressed hesitation toward incorporating new technological advancements into their daily routines. In addition, there was apprehension about using the chat forum stemming from previous negative experiences [108]. Concerns included the potential for the chat to focus excessively on individual issues or lead to cyberbullying based on caregivers’ past encounters with other social media platforms [108].

#### Theme 3: Suggestions to Improve IT Applications

##### Overview

This theme explores the proposed improvements to and adaptations of IT applications aimed at addressing the specific needs and challenges faced by informal caregivers. These are improvements in the user interface, navigation, and language; personalization and customization; integration with existing IT services; additional functions in IT applications; and IT support and training. A total of 56% (22/39) of the articles reviewed were classified under in this theme.

##### Improvements in User Interface, Navigation, and Language

In several studies (12/39, 31%), enhancements to the user interface, navigation, and language were suggested for system improvement [84,89,90,93,101,104,106,107,112,114,117,118]. This involved maintaining consistent and commonly used menu icons, ensuring uniform language across the system for effective communication with end users [85,118], and implementing plain icons with hover-over features to enhance system responsiveness [104]. These adjustments aimed to improve user experience and make the system more intuitive and accessible. The caregivers also suggested adding visual aids, warnings for potentially distressing content, and features such as frequently viewed buttons for easier navigation [116]. They also proposed rolling discussion topics and emoji reactions to enhance engagement with the forum [116]. Moreover, customization options were recommended for a more personalized user experience [106]. Caregivers recommended incorporating introductory text for created content, including videos and educational materials [108]. This serves to provide context and enhance the understanding of the content, offering users a more informed and engaging experience.

##### Personalization and Customization

Caregivers expressed a strong desire for personalized and customized IT applications that aligned with their specific needs and use context [81,84,89,93,104,106,112,114,117]. For web-based family support systems to be truly effective, it is essential to introduce them promptly and tailor them to the individual preferences of working caregivers, accommodating each caregiver’s unique circumstances [93]. A persistent concern voiced by participants was the importance of maintaining a sense of ownership during caregiving, particularly when they felt that their caregiving role dominated other aspects of their lives, such as being a partner, parent, or sibling [117]. The suggestion was to shift the focus from designing solely for the caregiver as a user to exploring designs that consider caregivers with diverse roles and responsibilities, such as managing a career or caring for young children. The goal was to develop online support that enables caregivers to preserve their identity, emphasizing the broader spectrum of roles they fulfill rather than exclusively focusing on their caregiving responsibilities [117].

##### Integration With Existing IT Services

Caregivers and patients use various IT services, including health care journals, throughout the caregiving journey. The caregivers expressed a desire for seamless integration between existing services and the proposed ones [111,116]. Specifically, linking CareHeroes (a proposed IT application) with other telehealth technologies, such as monitoring blood pressure, blood sugar, and mobility, was recommended for better usability [111]. This integration was anticipated to substantially amplify the platform’s potential impact, creating a more comprehensive and interconnected health care ecosystem [111].

##### Additional Functions in IT Applications

Several studies (3/39, 8%) suggested additional features for IT applications. Caregivers noted the need for multiple accounts to allow various family members and friends to participate in caregiving through the application, enabling a collaborative approach [90,105,106]. They also requested notifications and reminders for tasks but were concerned that automated reminders might feel “generic” and overwhelming. Customizable, personalized reminders were recommended to improve the user experience [106,107]. Caregivers suggested customizing messages in reminders, allowing for personalized and context-specific notifications [107]. Caregivers also struggled to find information within some applications, prompting the suggestion of a search function to enhance content accessibility and navigation [96,109,114].

##### IT Support and Training

Caregivers strongly desired easily accessible IT support and training as they encountered various issues while using IT applications [80,83,96,101,110,111,118]. To overcome these issues, caregivers recommended providing education and training on computer and internet use coupled with technical support for working caregivers [93]. The proposal included the addition of a “support” section in the main menu, enabling participants to request technical or emotional support directly from the application [118]. To enhance understanding of IT applications, instructional videos were recommended to educate caregivers about specific challenging tasks [111]. Furthermore, digital skill training was emphasized, particularly for caregivers with less experience with web services [110]. These comprehensive measures aimed to empower caregivers with the necessary support and knowledge to navigate and use IT applications effectively.

## Discussion

### Principal Findings

In this study, of the 82 selected articles, we examined 44 (54%) that presented design recommendations for IT applications for informal caregivers. Six overarching themes emerged: optimizing information access, support systems for informal caregivers, streamlining care-related tasks for informal caregivers, fostering informal caregivers’ well-being, communication with formal health care professionals, and task support. While these themes provide distinct categories, there are instances in which they overlap, indicating interconnections and complementary aspects among these identified areas. These design recommendations can contribute to further design of support applications for informal caregivers. Most of the included studies (44/82, 54%) on design used qualitative methods, including interviews and focus groups with caregivers, whereas some (5/44, 11%) also used mixed methods. Some of the included design studies (14/44, 32%) were focused on dementia caregivers, but we also found a mix of informal caregivers for cancer, older adults, stroke, and children with medical complexities. In this study, we also examined the perceptions and evaluations of IT applications among informal caregivers, analyzing 48% (39/82) of the selected studies in this category, which evaluated IT applications. In total, 3 broad themes emerged: facilitators of using IT applications, barriers to using IT applications, and suggestions to improve IT applications for informal caregivers. Interestingly, all the studies uniformly indicated a positive reception among informal caregivers regarding the use of IT applications. Most studies (28/39, 72%) used qualitative interviews for the evaluation of IT applications, with some (11/39, 28%) using mixed methods. Qualitative approaches provided detailed insights into caregivers’ contexts and preferences. Some studies from the medical field (4/39, 10%) used randomized controlled trials, which offered systematic assessments of the broader medical impacts of IT applications on both caregivers and patients. While many evaluation studies (18/39, 46%) were focused on mental impairments, a notable gap exists in research addressing caregivers dealing with various physical diseases, suggesting a potential area for future exploration.

One major strength of this review is its comprehensive search strategy, spanning diverse fields such as information systems, human-computer interaction, software development, health informatics, and health care. The identified facilitators of using IT applications, barriers to using IT applications, and improvements for IT applications for informal caregivers provide a foundation for further in-depth exploration. In total, 2 authors independently conducted the analysis, subsequently engaging in discussions to enhance the credibility of this review and reduce the risk of potential biases.

### Designing IT Applications for Informal Caregivers

Tailored education, training, and accessible resources are important in assisting informal caregivers [68,122]. Providing this in an appropriate format may reduce the considerable burden on informal caregivers. Recent studies emphasize the importance of curated information, ensuring that caregivers access relevant and easily understandable content [123,124]. However, these information needs vary throughout the caregiving journey [125,126]. Integrating evidence-based insights into these applications is an essential aspect of supporting caregivers effectively [63,64].

Community and peer support play significant roles in caregivers’ well-being [8,127]. Recent findings emphasize the value of shared experiences and connections among informal caregivers, providing emotional reinforcement and practical guidance [128,129]. The literature also suggests that feeling part of a community can help reduce the social isolation that many caregivers may experience [130]. Testimonials and shared experiences within caregiving communities foster confidence and a sense of solidarity among caregivers [131].

Efforts to streamline caregiving tasks encompass various dimensions crucial for effective support systems [132]. Coordinating appointments, treatments, and caregiving responsibilities has been identified as pivotal for optimizing care provision [133]. This process involves various activities, including communication, monitoring, and information sharing, and is influenced by patient-centeredness [134]. Effective care coordination interventions have been shown to benefit patients with specific conditions, particularly chronic conditions [135]. Informal caregivers play a crucial role in managing the health of older adults, particularly in medication management [136,137]. Monitoring technologies can enable caregivers to provide more care without increasing burden [138]. However, the impact of these changes on caregivers’ health and well-being is poorly understood [1].

Recent studies have highlighted the potential of IT applications for supporting caregiver well-being by reducing caregiver burden and stress [139]. Lorca-Cabrera et al [140] and Lam and Lam [141] highlighted the positive effects of web-based interventions and internet use on caregiver well-being, including improved mental health and reduced anxiety and distress. The literature also indicates the importance of IT applications for improving patients’ and their caregivers’ quality of life [28,142]. These IT interventions are known to reduce caregivers’ stress and depression [29].

Our findings suggest that informal caregivers benefit from communication with formal health care professionals. Effective communication between formal health care professionals and informal caregivers is crucial for the well-being of care recipients and the reduction of caregiver burden [143]. However, this communication is often hindered by a lack of clarity on the informational needs of caregivers [144], confusion about available services [145], and a lack of consideration for the views of informal caregivers [146]. The use of technology to mediate this communication has shown potential, particularly in aged care [43]. In the context of dementia care, formal caregivers face challenges in communication due to the impairments of care recipients [147]. Robust communication channels between caregivers and health care entities significantly impact caregiving experiences [147]. Enabling access to health care information, formal support coordination, and guidance from health care professionals is crucial for informed decision-making and ensuring optimal care provision.

### Evaluation of IT Applications for Informal Caregivers

Our findings from the studies evaluating the use of IT applications indicate that informal caregivers indeed valued the usefulness of these applications. However, they voiced concerns about the ease of use associated with these applications, in line with previous research [23,148]. A recent scoping review on the evaluation of smartphone apps for informal caregivers of patients with chronic illnesses also indicated that IT applications could play a significant role in informal caregiving, and many of the apps found in this review provided new opportunities for caregivers to access health information [22]. In another systematic review focusing on the implementation of IT applications for informal caregivers of individuals with dementia, it was determined that these interventions have the potential to significantly improve the well-being of caregivers [149]. Furthermore, by empowering caregivers to offer more effective and sustained care to patients, these eHealth interventions hold promise for reducing future burdens on health care services [149]. The importance of caregivers’ well-being was evident in our findings as well. Caregivers highly valued functions such as online forums, video meetings, and educational content. They highlighted how these features provided valuable information and support, contributing significantly to the enhancement of their psychological well-being.

Our findings showed that the most identified facilitators of using IT applications for caregivers were related to caregivers’ individual needs for personalized information, social support, education, and training to support their caregiving responsibilities. The information, education, and training should be personalized according to the caregivers’ situation, their specific caregiving needs, and the care recipient’s medical condition. Personalization and customization demand a user-centered and holistic approach to designing IT applications; active and early involvement of users is necessary to create applications that are tailor-made according to their needs [23,150].

It is noteworthy that the facilitators of using IT applications for caregivers, as well as the suggestions for improvement, were in line with the design recommendations gathered in this study. This alignment suggests that caregivers appreciate the implementation of design recommendations in IT applications when they use them. Most of the design recommendations were derived from the potential users of these applications. This underscores the significance of adopting a user-centered approach in which involving users in the requirement-gathering process significantly enhances the usability of these applications [148,151].

### Limitations and Future Research

Certain constraints related to this review necessitate consideration. Only 1 reviewer conducted the article identification, screening of titles, and data extraction for this review. Hence, there is a possibility that some relevant articles were not identified. Another limitation is that the review’s broad nature limits the in-depth exploration of specific themes. Future research could focus on providing more detailed insights into the identified themes. Future research could also focus on a review that compares the IT application requirements of informal caregivers with those of formal caregivers. This could provide a broader context for researchers. While our review included a few articles that dated back a decade (10/82, 12%), the core design recommendations and evaluations of use by informal caregivers remained relatively stable over time. This is because the fundamental principles guiding caregiver support and design considerations have not drastically changed, and these studies still offered relevant insights into these principles. In addition, while adhering to a stringent scoping review structure, there remains a possibility of overlooking pertinent research, particularly when searching through an extensive array of evidence, including gray literature. The studies often lacked diversity. Many studies primarily included women, potentially excluding experiences of male caregivers. Where reported, studies often lacked representation of various ethnicities and socioeconomic backgrounds, suggesting that the findings might not apply equally to diverse caregiver populations. Due to the self-selecting nature of participants in many reviewed studies, the findings might not represent all family caregivers. This is because individuals who self-select are often more motivated and actively engage in using IT applications. Consequently, caregivers facing different challenges or lacking those specific motivations might have been unintentionally excluded. This limitation raises concerns about the generalizability of the review’s conclusions to the broader caregiver population.

### Conclusions

This review is the first to explore the design recommendations and evaluation of the use of IT applications for informal caregivers. It provides a summary of design recommendations to begin the design of an IT application for informal caregivers. It also provides a compilation of facilitators of using IT applications, barriers to using IT applications, and suggestions for improving IT applications for informal caregivers based on previous literature. This review was based on 82 articles, of which 44 (54%) were focused on design recommendations and 39 (48%) were focused on evaluations of the use of IT applications for informal caregivers. Six overarching themes emerged related to designing IT applications: information access, support systems, streamlining care-related tasks, fostering informal caregivers’ well-being, communication with formal health care professionals, and task support. Furthermore, 3 broad themes emerged related to the evaluation of IT applications for informal caregivers: facilitators of using IT applications, barriers to using IT applications, and suggestions for improving IT applications for informal caregivers. These findings provide a road map for enhancing user-centric IT applications in informal caregiver support technologies.

## Appendix

Multimedia Appendix 1 PRISMA-ScR (Preferred Reporting Items for Systematic Reviews and Meta-Analyses extension for Scoping Reviews) fillable checklist.

Multimedia Appendix 2 Search strategy.

Multimedia Appendix 3 Summary table of included articles.

## Abbreviations

PICOS: population, intervention, comparator, outcome, and study design
PRISMA-ScR: Preferred Reporting Items for Systematic Reviews and Meta-Analyses extension for Scoping Review
RQ: research question

## Data Availability

All data generated or analyzed during this study are included in this published article (and its supplementary information files).
