# Supplementary material for: Designing and Evaluating IT Applications for Informal Caregivers: Scoping Review
Source: J Med Internet Res. 2024 Oct 23;26:e57393. doi: 10.2196/57393 (PMC11541158; doi:10.2196/57393)
Supplement: Multimedia Appendix 2 [file jmir_v26i1e57393_app2.docx]

**Search strategy**

***Keyword search groups for database search***

(1) Informal caregivers (e.g., caregiver, caregiver, spouse, and partner)

(2) IT applications (e.g. internet, app, or eHealth)

(3) Design and evaluation (e.g., design, evaluation, needs, usability or experience).

The keywords were applied on metadata. Librarians were consulted when constructing the search string.

| Keywords | Search words |
| --- | --- |
| Group 1: informal caregiver | “Home nursing” OR “informal care*” OR “family care*” |
| Group 2: IT solution | “Mobile application*” OR “ICT solution*” OR “ICT” OR eHealth OR “e-coaching system*” OR “*coaching system*” OR “digital solution*” OR “IT solution*” OR “internet-based interventions*” OR telehealth* |
| Group 3: design or evaluation | Design OR evaluation OR effectiveness OR usability OR requirements OR needs OR perspective OR “user experience*” |

***Keyword search groups for manual Google Scholar search***

Google Scholar search was conducted using the keyword field in Publish or Perish (Version: 8.9.4538). Multiple combinations with keywords from each search group were used for the Google Scholar search on metadata and these four combinations gave the most relevant results.

Each of the following search string was searched separately and 100 hits were collected from each search:

(1) family carer design OR evaluation “internet intervention”

(2) family carer design OR evaluation ICT

(3) family carer design OR evaluation "digital intervention"

(4) family carer design OR evaluation application.
