# Supplementary material for: Designing and Evaluating IT Applications for Informal Caregivers: Scoping Review
Source: J Med Internet Res. 2024 Oct 23;26:e57393. doi: 10.2196/57393 (PMC11541158; doi:10.2196/57393)
Supplement: Multimedia Appendix 3 [file jmir_v26i1e57393_app3.docx]

| Authors | Study type | Study design | Condition cared for | Study objective(s) | Study location |
| --- | --- | --- | --- | --- | --- |
| Rathnayake et al., 2021 [39] | Design | Mixed method | Dementia | To develop an mHealth application for family caregivers of people with dementia to address functional disability care needs. | Australia |
| Macaden et al., 2021 [40] | Design | Survey | Dementia | To develop the symptom-monitor and track feature within the CogniCare app to support family carers of people living with dementia | UK |
| Lobão et al., 2021 [41] | Design | Qualitative | Dementia | To develop an ICT solution to support informal caregivers of patients with dementia | Portugal |
| Leslie et al., 2021 [42] | Design | Qualitative | Older adults | Focuses on informal caregivers’ perspectives on the role of information and communications technology (ICT) in supporting these goals. | Canada |
| Dickman Portz et al., 2020 [43] | Design | Qualitative | Palliative Care | To identify recommendations for tools, resources, and functionality needed to create useful palliative care mobile apps for patients and families | USA |
| Nurgalieva et al., 2019 [44] | Design | Qualitative | Older adults | Evaluate design alternatives that could mediate sharing of health and wellbeing information (HWBI) in nursing home care context | Italy |
| Lederman et al., 2019 [45] | Design | Qualitative | Young people with mental illness | To demonstrate how a technology-mediated mental health therapy, incorporating psycho-education, therapist moderation, and social networking, supports caregivers of young people with mental illness. | Australia |
| Heynsbergh et al., 2019 [46] | Design | Qualitative | Cancer | To design a smartphone app prototype for carers of adults with cancer. | Australia |
| Chaar et al., 2019 [47] | Design | Qualitative | Hematopoietic Stem Cell Transplant | Explored the views and perceptions of family caregivers of patients undergoing HCT and their input regarding further technology development and expansion of BMT Roadmap into the outpatient setting (referred to as Roadmap 2.0). | USA |
| Allemann et al., 2019 [48] | Design | Qualitative | Heart failure | To explore the perceptions of IT solutions as supportive aids among family members of persons with Heart failure | Sweden |
| McNaney et al., 2017 [49] | Design | Qualitative | Dementia | To explore how technology can support younger family members in their interactions with people with dementia. | UK |
| Tixier and Lewkowicz 2016 [50] | Design | Qualitative | General | To explore the types of social support valued by informal caregivers of older adults, focusing on their experiences and preferences to address the isolation and challenges they face. | France |
| Schorch et al., 2016 [51] | Design | Qualitative | General | To understand the care practices and needs of informal caregivers, particularly relatives caring for chronically ill individuals at home, in order to better support their work and coordination. | Germany |
| Bosch and Kanis 2016 [52] | Design | Qualitative | General | Highlight key opportunities for technology design for informal caregivers who provide long-term in-home care. | The Netherlands |
| Meiland et al., 2014 [53] | Design | Qualitative | Dementia | To inventory the needs and wishes of informal caregivers and care recipients regarding the development and design of the new integrated Rosetta system and to describe the system to be developed. | The Netherlands |
| Mchugh et al., 2014 [54] | Design | Qualitative | Dementia | To explore how information and communication technology (ICT) can help reduce the burden on informal caregivers of persons with dementia by addressing their support needs, social isolation, and the caregiver-patient relationship. | Ireland |
| Hwang et al., 2012 [55] | Design | Qualitative | Older adults with Dementia (OAWDs) | To involve informal caregivers in the needs analysis and design of smart home user interfaces, focusing on the intelligent COACH system that assists older adults with dementia in daily activities. The goal is to alleviate caregiver burden and promote independent living for dementia patients. | Canada |
| Renyi et al., 2018 [56] | Design | Mixed method | General | to identify the needs and requirements for mobile collaboration support in informal care networks to develop a user-centered design for collaboration software tools. | Germany |
| Moberg et al., 2022 [57] | Design | Qualitative | Dementia | To explore stakeholders’ perspectives on a potential mobile application through which family caregivers could be supported by healthcare professionals in caring for a person with dementia living at home. | Sweden |
| Ahmad et al., 2023 [58] | Design | Qualitative | Head and Neck Cancer | To explore the situation and context of informal caregivers of patients with HNC and their needs for designing and developing a web-based intervention (Carer eSupport). | Sweden |
| Premanandan et al., 2023 [59] | Design | Qualitative | General | To identify the unmet needs of caregivers in Sweden and provides design suggestions for an e-coaching application using the persuasive system design (PSD) model. | Sweden |
| Premanandan et al., 2023 [8] | Design | Qualitative | General | This study aims to investigate the needs of immigrant informal caregivers in Sweden and discuss the application of the Persuasive System Design Model (PSDM) to develop an e-coaching prototype. | Sweden |
| Molinari-Ulate et al., 2023 [60] | Design | Qualitative | Dementia | to culturally adapt and co-design the iSupport online training and support program for informal caregivers of people living with dementia in Castilla y León, Spain, to enhance e-health intervention accessibility and effectiveness in remote and rural areas. | Spain |
| Ciuffreda et al., 2023 [61] | Design | Qualitative | Older adults | To develop and evaluate the GUARDIAN ecosystem, a support system for older adults and their caregivers in indoor living environments. |  |
| Gris et al., 2023 [62] | Design | Qualitative | Dementia | To explore their needs and preferences of caregivers to inform the co-design of the DemiCare integrated system. | Italy |
| Haji Mukhti et al., 2022 [63] | Design | Qualitative | General | To explore the need for a mobile application in stroke management by informal caregivers of stroke patients. | Malaysia |
| Fan et al., 2023 [64] | Design | Qualitative | Alzheimer | To characterize the challenges and needs of Alzheimer’s and Alzheimer's disease-related dementia family caregivers to inform the design of an internet-based, artificial intelligence-driven digital resource platform. | USA |
| Egan et al., 2021 [65] | Design | Qualitative | General | To co-design a novel mobile application to support carers to undertake regular physical activity from home during and beyond COVID-19 restrictions. | UK |
| Giroux et al., 2019 [66] | Design | Qualitative | Older adults | To create an eHealth tool in collaboration with community organizations, health and social service professionals, and caregivers, aiming to enhance the early detection of needs among older individuals and optimize the utilization of existing resources. | Canada |
| Gutierrez and Ochoa 2021 [67] | Design | Qualitative | Older adults | To understand and support informal elderly caregiving in Southern Cone families through a multi-method approach, defining roles, outlining concerns via a caregiving matrix, and designing contextualized strategies using computer-supported technology. | South Cone |
| Hashemi et al., 2018 [68] | Design | Qualitative | Cancer | To identify the unmet needs of the caregivers of end-of-life cancer patients. | Iran |
| Köhle et al., 2015 [69] | Design | Qualitative | Cancer | To investigate the interest of caregivers in a web-based psychological intervention and to determine their requirements and preferences for such an intervention. | The Netherlands |
| Martínez-Alcalá et al., 2017 [70] | Design | Qualitative | Older adults | To introduce and validate the eCuidador platform, which provides cloud-based services to support primary caregivers, aiming to improve their health and quality of life. | Mexico |
| Masterson-Algar et al., 2022 [71] | Design | Qualitative | Dementia | Discusses the co-design of an adapted version of the iSupport e-health training programme for young dementia carers. | UK |
| Moreno-Cámara et al., 2019 [72] | Design | Qualitative | Dementia | The aim of this study was to identify, categorize, and examine the perceived requirements of caregivers of elderly individuals with dementia throughout the caregiving process. | Spain |
| Sepehri et al., 2023 [73] | Design | Qualitative | Children living with health complexity | To understand the needs of parent caregivers of Children Living with Health Complexity (CLHC) through a co-design process. | Canada |
| Shreve et al., 2016 [74] | Design | Qualitative | Dementia | To identify which information technology design characteristics and functionalities would be most beneficial for family caregivers of adults with Alzheimer’s disease or other dementias. | USA |
| Siddiqui et al., 2023 [75] | Design | Qualitative | Severe mental illness | To explore how caregivers in under-resourced settings in India can be better supported through everyday digital technologies. | Australia |
| Vaughan et al., 2018 [76] | Design | Qualitative | Wounded, ill, and injured in military service; members and veterans | Investigate the usage and attitudes towards an online social support program designed for informal caregivers of wounded, ill, and injured military service members and veterans in the United States. | USA |
| Williamson et al. 2014 [77] | Design | Qualitative | Older adults | To assess the information and technology needs of long-distance caregivers (LDCs) through semi-structured interviews and prototype demonstrations. | USA |
| Liverpool and Edbrooke-Childs 2021 [78] | Mixed methods | Qualitative | Child and adolescent mental health services (CAMHS) | To outline the creation of a digital decision support intervention, grounded in evidence and theory, for parents and caregivers of youth accessing CAMHS. | UK |
| Xu et al., 2020 [79] | Design | Qualitative | Atopic Dermatitis | To investigate the preferred features and content of a smartphone application aimed at facilitating atopic dermatitis self-care for informal caregivers. | Singapore |
| Ducharme et al. 2014 [80] | Mixed methods | Qualitative | Dementia | To record the support needs that have not been fulfilled for this particular group of caregivers for creating new IT interventions. | Canada |
| Wan et al. 2021 [81] | Design and Evaluation | Mixed method | Colorectal Cancer | To describe the development of a smartphone-based interactive CRC self-management program called iCanManage, in order to improve health outcomes for patients undergoing elective colorectal cancer surgeries and their family caregivers. | Singapore |
| Cheng et al., 2020 [82] | Design and Evaluation | Qualitative | Children with medical complexity | To design and test the usability of a mobile application to support family-delivered enteral tube care for children with medical complexity | USA |
| Costa Stutzel et al., 2019 [83] | Evaluation | Qualitative | Older adults with chronic diseases | Assessing Mobile System for Elderly Monitoring’s (SMAI) usability, functionality usage patterns, and qualitative perceptions among users to determine its effectiveness in improving caregiver support and facilitating healthcare team communication. | Brazil |
| Xiao et al., 2013 [84] | Evaluation | Qualitative | Older adults | To evaluate web interfaces for a sensor-based home monitoring system designed for informal caregivers of independently living seniors. | Canada |
| Renati et al., 2022 [85] | Evaluation | Qualitative | Substance abuse | To evaluate the usability of a mobile health application (mHealth app) designed for caregivers of individuals with substance use disorders. | Italy |
| Thompson et al., 2023 [86] | Evaluation | Qualitative | Dementia | To validate training material for the MATCH mobile application, which trains family caregivers in using music therapy to support people living with dementia. | Australia |
| Păsărelu et al., 2023 [87] | Evaluation | Qualitative | ADHD | To evaluate the usability of a mobile app, ADHD Coping Card, designed for parents of children with ADHD. | Romania |
| Dam et al., 2017 [88] | Evaluation | Qualitative | Dementia | Assessment of an online social support intervention named Inlife’s feasibility and the methods used to gauge its effectiveness. | The Netherlands |
| Wilding et al., 2021 [89] | Evaluation | Qualitative | Dementia | To co-design a website/mobile app and Zoom videoconferencing tool for carer of rural people living with Dementia. | Australia |
| Teles et al., 2021 [90] | Evaluation | Mixed method | Dementia | Evaluate the usability of the European-Portuguese version of iSupport | Portugal |
| Gomes et al., 2022 [91] | Evaluation | Qualitative | General | To evaluate the usability of the web and mobile applications of the Help2Care platform | Portugal |
| Quinn et al., 2019 [92] | Evaluation | Qualitative | Older adults | To determine the usability of a mobile app in a community-based older adult population | USA |
| Brouns et al., 2018 [93] | Evaluation | Qualitative | Stroke | To investigate the factors that affect the adoption of eRehabilitation in stroke recovery, focusing on stroke patients, informal caregivers, and healthcare professionals. | The Netherlands |
| Andersson et al., 2017 [94] | Evaluation | Qualitative | Older adults | To explore the perceived benefits and challenges of ‘A Good Place’ for supporting working informal carers who provide unpaid family care for older relatives. | Sweden |
| Kajaks et al., 2015 [95] | Evaluation | Qualitative | General | To assess the SafeBack mobile application's usability and identify areas for improvement to enhance its effectiveness as a posture training and injury prevention tool for caregivers. | Canada |
| Lundberg 2014 [96] | Evaluation | Qualitative | Dementia or Stroke | to better understand how IT applications can support elderly informal caregivers of dementia or stroke patients. | Sweden |
| Blusi et al., 2014 [97] | Evaluation | Mixed method | Older adults | To compare the benefits of e-health caregiver support versus conventional caregiver support among rural family caregivers using a mixed methods approach. | Sweden |
| Blusi et al., 2013 [98] | Evaluation | Qualitative | General | To explore the impact of ICT-based caregiver support on older family carers in rural areas caring for spouses at home. | Sweden |
| Chiu and Eysenbach 2011 [99] | Evaluation | Qualitative | Dementia | To enhance understanding of how family caregivers utilize a Web-based intervention support | China |
| Torp et al., 2008 [100] | Evaluation | Mixed method | Older adults | To investigate whether the utilization of IT by informal caregivers of frail elderly individuals would facilitate increased knowledge about chronic illness, caregiving strategies, and coping mechanisms. | Norway |
| Andersson et al., 2002 [101] | Evaluation | Qualitative | Older adults | To evaluate the usability of the ACTION application for informal caregivers | Sweden |
| Husebø 2021 [102] | Evaluation | Qualitative | Colorectal Cancer (CRC) | To investigate the perspectives of CRC patients, their informal caregivers, and healthcare professionals (HCPs) regarding IT. | Norway |
| Hermaszewska and Sin 2021 [103] | Evaluation | Qualitative | Autism | To identify the need for online interventions, defining content and design preferences, and outlining strategies for effective implementation. | UK |
| Rottenberg and Williams 2021 [104] | Evaluation | Mixed method | Older adults | To assess how informal caregivers of older adults perceived the web-based delivery of the Caregiving Essentials course. | Canada |
| Egan et al., 2022 [105] | Evaluation | Qualitative | Physical health | To assess a newly developed Carefit designed to improve physical activity among informal caregivers. | UK |
| Lam et al., 2022 [106] | Evaluation | Qualitative | Stroke | To explore user expectations and the factors that promote or hinder the adoption of a ‘virtual clinic’ for stroke survivors and caregivers. | Hong Kong |
| Boutilier et al., 2022 [107] | Evaluation | Mixed method | Dementia | To assess the feasibility of CareVirtue by examining the patterns of usage, caregivers' perceptions of its acceptability, and factors influencing its use. | USA |
| Kwok et al., 2023 [108] | Evaluation | Mixed method | Dementia | To pilot test a socially augmented, self-directed version of the positive emotion regulation intervention, Social Augmentation of Self-Guided Electronic Delivery of the Life Enhancing Activities for Family Caregivers (SAGE LEAF). | USA |
| Tibell et al., 2022 [109] | Evaluation | Qualitative | Life threatening illness | To assess the feasibility of a web-based intervention, "narstaende.se," as perceived by spouses of patients receiving specialized home care. | Sweden |
| Austrom et al., 2015 [110] | Evaluation | Qualitative | Dementia | To pilot a web-based videoconference support group for dementia caregivers, assessing experiences, and evaluating impacts on caregiver anxiety, depression, physical health, and self-efficacy in managing caregiving challenges. | USA |
| Barbabella et al., 2018 [111] | Evaluation | Mixed method | Older adults | To assess the usage and usability of the InformCare psychosocial Web-based program among informal caregivers of older people in Italy, Sweden, and Germany. | Italy, Germany and Sweden |
| Brown et al., 2016 [112] | Evaluation | Mixed method | Alzheimer and or other forms of Dementia | To evaluate the adoption, effectiveness, and improvement areas CareHeroes designed for caregivers of individuals with Alzheimer’s disease or other forms of dementia. | USA |
| Crotty et al., 2020 [113] | Evaluation | Qualitative | Cancer | To investigate the unaddressed information management requirements of informal caregivers. | USA |
| Iribarren et al., 2019 [114] | Evaluation | Qualitative | Alzheimer’s Disease and Related Dementias (ADRD) | To determine the information and communication requirements of Hispanic family caregivers for individuals with Alzheimer’s Disease and Related Dementias (ADRD), and explore how online tools can address these needs. | USA |
| Ploeg et al., 2018 [115] | Evaluation | Qualitative | Older adults with ADRD and multiple chronic conditions | To assess the impact of a self-administered, psychosocial, supportive, Web-based Transition Toolkit (My Tools 4 Care - MT4C) on caregivers of older adults with ADRD and multiple chronic conditions, identify the most and least beneficial features of MT4C, and gather recommendations for improvements to the toolkit. | Canada |
| Rettinger et al., 2020 [116] | Evaluation | Mixed method | Dementia | To evaluate the satisfaction, technical performance, and usability of the Android application ‘DEA’ designed to support informal caregivers of people with dementia. | Austria |
| Batchelor et al., 2022 [117] | Evaluation | Qualitative | Psychosis | To investigate caregivers' experiences with COPe-support (Carers for People with Psychosis e-support) on its acceptability, its impact on their well-being and caregiving, and their suggestions for enhancing COPe-support. | UK |
| Honary et al., 2018 [118] | Evaluation | Qualitative | Psychosis or bipolar disorder | To employ a user-centered design approach in evaluating a user-friendly web-based intervention, adapted from the Relatives Education And Coping Toolkit (REACT) booklet, to meet the informational and emotional needs of relatives caring for individuals with psychosis or bipolar disorder. | UK |
| Sin et al., 2019 [119] | Evaluation | Mixed method | Psychosis | To assess usability, system heuristics, and perceived acceptability in a usability study aimed at determining the appropriateness of the intervention prototype for the intended user group. | UK |
